# The CAR T‐Cell Mechanoimmunology at a Glance

**DOI:** 10.1002/advs.202002628

**Published:** 2020-11-03

**Authors:** Rui Li, Chao Ma, Haogang Cai, Weiqiang Chen

**Affiliations:** ^1^ Department of Mechanical and Aerospace Engineering New York University Brooklyn NY 11201 USA; ^2^ Department of Biomedical Engineering New York University Brooklyn NY 11201 USA; ^3^ Tech4Health institute NYU Langone Health New York NY 10016 USA; ^4^ Department of Radiology NYU Langone Health New York NY 10016 USA; ^5^ Laura and Isaac Perlmutter Cancer Center NYU Langone Health New York NY 10016 USA

**Keywords:** CAR T‐cell, immunological synapse, immuno‐oncology, mechanoimmunology, mechanotransduction

## Abstract

Chimeric antigen receptor (CAR) T‐cell transfer is a novel paradigm of adoptive T‐cell immunotherapy. When coming into contact with a target cancer cell, CAR T‐cell forms a nonclassical immunological synapse with the cancer cell and dynamically orchestrates multiple critical forces to commit cytotoxic immune function. Such an immunologic process involves a force transmission in the CAR and a spatiotemporal remodeling of cell cytoskeleton to facilitate CAR activation and CAR T‐cell cytotoxic function. Yet, the detailed understanding of such mechanotransduction at the interface between the CAR T‐cell and the target cell, as well as its molecular structure and signaling, remains less defined and is just beginning to emerge. This article summarizes the basic mechanisms and principles of CAR T‐cell mechanoimmunology, and various lessons that can be comparatively learned from interrogation of mechanotransduction at the immunological synapse in normal cytotoxic T‐cell. The recent development and future application of novel bioengineering tools for studying CAR T‐cell mechanoimmunology is also discussed. It is believed that this progress report will shed light on the CAR T‐cell mechanoimmunology and encourage future researches in revealing the less explored yet important mechanosensing and mechanotransductive mechanisms involved in CAR T‐cell immuno‐oncology.

## Graphical abstract

This progress report summarizes how chimeric antigen receptor (CAR) T‐cell orchestrates multiple mechanical forces through the immunological synapse formed between the T‐cell and target cancer cell to successfully commit cytotoxic immune function in immunotherapy. An understanding of the CAR T‐cell mechanoimmunology will shed light on the future design of safe yet efficient CAR T‐cells and mechanomedicines from a mechanoimmunological perspective.

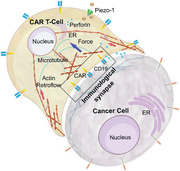

## 1 Introduction

**Figure 1:**
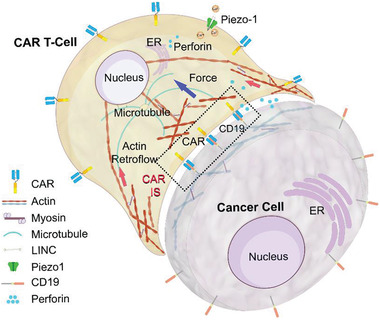
The nonclassical CAR T‐cell immunological synapse. CAR T‐cell orchestrates multiple mechanical forces through a dynamically organized macromolecular membrane assembly, the immunological synapse, formed between an activated T‐cell and a target cancer cell to successfully commit cytotoxic immune function.

Chimeric antigen receptor (CAR) T‐cells are highly effective, genetically engineered cytotoxic T‐cells that combine both antigen‐binding and T‐cell activating functions into a single receptor for cancer therapy.^[^ ^1^ ^]^ Cluster of differentiation 19 (CD19)‐targeted CAR T‐cell immunotherapy has emerged as a promising Food and Drug Administration (FDA)‐approved adoptive T‐cell therapy for the treatment of B‐cell malignancies.^[^ ^2^ ^]^ During cancer immunotherapy, CAR T‐cell orchestrates multiple mechanical forces through the immunological synapse (IS), a dynamically organized macromolecular membrane assembly formed between an activated T‐cell and a target cancer cell, to successfully commit cytotoxic immune function (**Figure** **1**).^[^ ^3^ ^]^ Such an immunologic process involves both a physical immunological handshake through an IS and a simultaneous bilateral transfer of soluble biochemical signals between the cytotoxic T‐cell and the cancerous cell. Both the CAR activation and IS formation are mechanotransductive processes that involve a spatiotemporal remodeling of the cytoskeleton and force transmission in the IS.^[^ ^4^, ^5^ ^]^ However, recent studies indicate that the mechanosensing and mechanotransductive mechanisms in CAR are largely distinct from the conventional T cell receptors (TCRs), and even more distinct among various engineered types of CARs.^[^ ^5^, ^6^ ^]^ Thus, a detailed understanding of the CAR T‐cell mechanoimmunological mechanisms is in urgent need yet remains less defined.

A hallmark of T‐cell cytotoxic activity is exerting mechanical forces through the IS and onto target cancerous cell and vice versa,^[^ ^7^ ^]^ whereas disturbances or loss of such forces greatly compromise CAR/TCR activation and cytotoxic function.^[^ ^7^, ^8^, ^9^ ^]^ As seen in the conventional T‐cell study, cytotoxic T‐cell expressing TCRs can recognize and bind specific antigen molecules of peptide‐bound major histocompatibility complex (pMHC) expressed on the target cancer cell. During the process, T‐cell is highly mechanosensitive and the TCR activation and force generation are regulated by the local mechanical cues^[^ ^10^, ^11^ ^]^ and geometric arrangement of binding ligands.^[^ ^12^ ^]^ Moreover, the force dynamically changes in the IS with a conformational transformation in the TCR–pMHC complex to guide the proper immune responses,^[^ ^13^, ^14^ ^]^ including rapid antigen sampling and serial secretion of lytic granules to the cancerous cells.^[^ ^15^, ^16^, ^17^ ^]^ Unlike the physiologic TCRs, CARs directly recognize tumor antigens independently of their expression of MHC antigens.^[^ ^1^ ^]^ These mechanosignals translated through a nonclassical IS formed at the interface between the CAR T‐cell and the target cell are also involved in CAR activation and CAR T‐cell cytotoxic function.^[^ ^5^ ^]^ A proper level of tension force is required to load on the CAR–antigen complex for an effective CAR activation. The cell‐driven cyclic filamentous actin (F‐actin) polymerization and depolymerization in cytoskeleton implicate the activation of mechanosensitive signaling and transmission of cellular forces during the IS formation and cytotoxic process.^[^ ^18^ ^]^ Essentially, the spatiotemporal kinetics of synaptic force in the IS largely determines the efficiency of antigen recognition and IS formation, and thus cytotoxic immune functions.^[^ ^19^ ^]^ Thus, maintaining the proper magnitudes and temporal dynamics of the mechanosignals in and through the IS will be essential for achieving an efficient CAR T‐cell in immunotherapy.^[^ ^20^ ^]^

CAR T‐cell mechanoimmunology has emerged as a highly interdisciplinary research field investigating the mechanosensing and mechanotransduction processes involved in the CAR recognition, activation, IS formation, and the T‐cell cytotoxic function.^[^ ^14^, ^21^ ^]^ Therefore, this progress report aims to condense the latest updates and major breakthroughs in CAR T‐cell mechanoimmunology research, and provide an insight on the evolutionary bioengineering strategies, platforms, and algorithms for investigating the currently explored and unexplored mechanoimmunological mechanisms. We anticipate that this progress report on CAR T‐cell mechanoimmunology will not only shed light on the future researches on the critical mechanosensing and mechanotransductive mechanisms in CAR T‐cell immuno‐oncology, but also pave the ways toward the design of next‐generation CAR T‐cells and the development of novel mechanomedicine solutions from a mechanoimmunological perspective.

## 2 The Fundamentals of CAR T‐Cell Mechanoimmunology

### 2.1 The Structure of Various Generations of CARs

**Figure 2:**
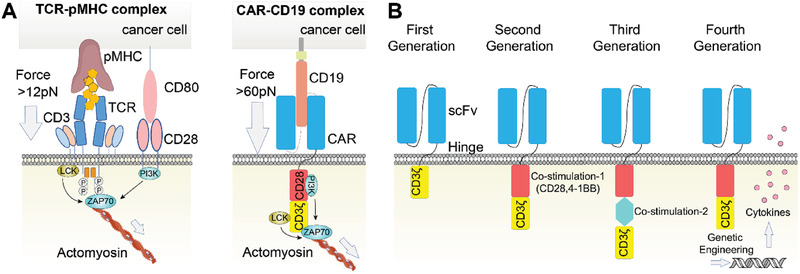
The schematic diagram of CAR structures. A) The different structural basis of TCR–pMHC and CAR–CD19 complexes. B) Different generations of CAR designs. Adapted with permission.^[^ ^39^ ^]^ Copyright 2019, Springer Nature.

CARs are recombinant transmembrane receptors composed of an extracellular binding domain of a monoclonal antibody for tumor‐associated antigen recognition, a hinge domain, and an intracellular signaling domain of a TCR molecule for initiating signal transduction that leads to T‐cell activation (**Figure** **2**).^[^ ^1^ ^]^ Similar to the treatment strategies employing monoclonal antibodies, T‐cells expressing CARs are highly targeted. More importantly, the CAR design can directly recognize tumor antigens independently of MHC expression by tumor cells, thus bypassing the MHC‐deficiency resulted immune evasion in conventional TCR recognition.^[^ ^38^ ^]^

Importantly, the costimulatory domains of CAR greatly impact the proliferation, effector functions, and persistence of CAR T‐cell. Identifying the specific effects and molecular mechanism of the emerging costimulatory domains is essential in designing the CARs. The first generation of CAR is made from antibody single‐chain variable fragments (scFvs) fused with a cytoplasmic CD3*ζ* signaling domain (Figure 2b).^[^ ^40^ ^]^ Such a CD3*ζ* cytoplasmic tail enables the CAR activation independent of pMHC, and the triggering of mechanosensitive signals in the CAR T‐cell. The combination of the costimulatory domains also finely tuned the cytotoxic activities of the CAR T‐cells. Considering the complementary roles of CD3 and CD28 in force generation,^[^ ^34^ ^]^ the second generation of CAR T‐cell commonly infuses a CD28 costimulatory domain proximal to the CD3*ζ* tail to strength the mechanotransduction of the CAR through the phosphatidylinositol‐3‐kinase (PI3K) signaling (Figure 2b).^[^ ^1^ ^]^ Clinical trials reported that CD28 effectively enhanced the mechanical signals generated in the immunoreceptors through PI3K pathway and promoted the proliferation of the cytotoxic CAR T‐cell with a high reaction rate to the tumor cells.^[^ ^34^ ^]^ Similar to CD28, the inducible T‐cell costimulator (ICOS) stimulates the CAR through a PI3K‐dependent mechanism to a more profound extent.^[^ ^41^ ^]^ Another well‐studied costimulatory ligand is the 4‐1BB, which prolongs the persistence of the cytotoxic CAR T‐cell. Thus, the third generation of CAR T‐cell incorporates both CD28 and 4‐1BB intracellular costimulatory domains in line to further increase the intensity and the persistence of mechanotransduction through the extracellular signal‐regulated kinase (ERK) signaling (Figure 2b).^[^ ^42^ ^]^ The 4‐1BB costimulation enhances the CAR T‐cell function and persistence through the NF‐*κ*B signaling, promotes T‐cell differentiation to central memory cells and protects against CAR T‐cell exhaustion, allowing for a long‐term elimination of tumor cells.^[^ ^43^ ^]^ Another third generation CD28‐OX40 CAR T‐cell shows a significant elevation in the production of IL‐2 and IL‐10 cytokines compared with that of the second generation CD28 or OX40 CAR T‐cell.^[^ ^44^ ^]^ The enhanced expression of IL‐2 and IL‐10 is beneficial for the effector functions and persistence of CAR T‐cells in vivo. The fourth generation of CAR T‐cell with single costimulatory domains (Figure 2b) trans‐genetically elevates the expression of cytokines like IL‐12 to propagate the intracellular mechanical cues from the costimulatory molecules like programmed cell death protein 1 (PD1), therefore promoting the CAR T‐cell killing efficiency.^[^ ^45^ ^]^ The single activation of CAR simultaneously triggers multiple endogenous mechanosignaling events and outputs including lytic granule release, cytokine secretion, and T‐cell proliferation.^[^ ^39^, ^46^ ^]^ In summary, an optimized selection of the costimulatory domains in the design of CAR and a clear understanding of the involved mechanobiological signals will show critical implications for the augment of the CAR T‐cell therapeutic performance.

### 2.2 Nonclassical Immunological Synapse of CAR T‐cell

**Figure 3:**
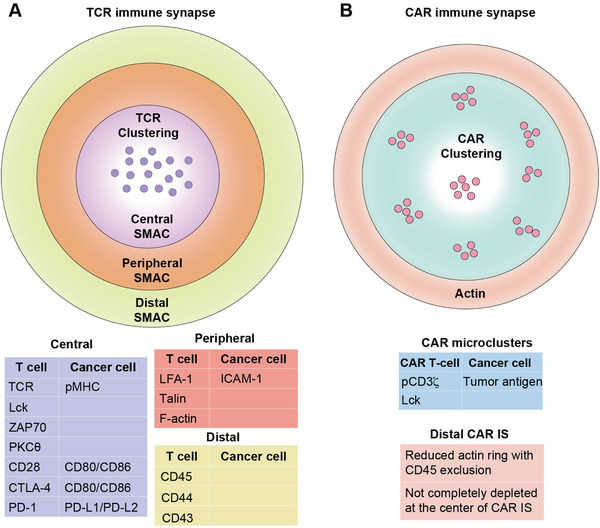
The classical TCR IS and nonclassical CAR IS. A) The classical TCR IS is comprised of three separate zones including the central, peripheral and distal supramolecular activation cluster (SMAC) zones, with distinct molecular components and functions. B) The nonclassical CAR IS has less organized structures, with diffusive CAR clustering and reduced actin distribution in the distal CAR IS. Adapted with permission.^[^ ^52^ ^]^ Copyright 2018, Frontiers Media S.A.

The activation and mechanotransduction of TCR and CAR rely on the respective formations of the classical and nonclassical IS (**Figure** **3**).^[^ ^38^ ^]^ During the formation of classical IS after TCR–pMHC ligation, there will be a sharp increase of the cortex in the contact zone, followed by the assembly of multiple actomyosin networks and the stabilization of the IS.^[^ ^47^ ^]^ The mature IS comprised three separate zones, including the central, peripheral and distal supramolecular activation cluster (SMAC) zones, with distinct immune functions (Figure 3A).^[^ ^48^ ^]^ Briefly, a distal actin ring composed of branched actin network allows the spreading of protrusions and generates retrograde flow in T‐cell to pass mechanical forces to the peripheral area. Many integrin molecules including lymphocyte function‐associated antigen‐1 (LFA‐1) localize in the peripheral area, and the activated integrins will bind to target cells to increase T‐cell adhesion. Such increased adhesion signals finally initiate the TCR signaling in the central area of the IS.^[^ ^49^ ^]^ The central IS remains relatively static and mainly translates the mechanical signals to the intracellular mechanocues on the cytoskeleton through the lymphocyte‐specific protein tyrosine kinase/zeta‐chain‐associated protein kinase 70 (Lck/ZAP70) complex.^[^ ^50^ ^]^ Finally, mechanotransduction in the classical IS induces the effector functions including the release of lytic granules and cytokines.^[^ ^8^ ^]^ In the late stage of effector functions, central actin networks would exert force to drive the detach of T‐cell from cancer cell,^[^ ^51^ ^]^ so that it ensures the long‐term serial cytotoxic effect on numerous cancerous cells.

Different from the organized zones in the classical IS, the nonclassical CAR IS has distinct, less organized structures and reduced cytoskeleton (Figure 3B).^[^ ^52^ ^]^ Just two minutes after contact, the multifocal protrusions in the CAR IS start to form and rapidly stimulate the downstream mechanosignaling and cytotoxic activities.^[^ ^38^ ^]^ Recently, Davenport et al. has compared the differences between the formations of a CAR IS and a TCR IS.^[^ ^38^ ^]^ They discovered that a CAR IS would be fully established within 5 minutes and form disorganized Lck patches, rather than the stable Lck clusters normally seen in a TCR IS. Such disorganized Lck patches lead to the smaller size of actin rings in the CAR IS compared to TCR IS, leading to a swift mechanotransduction in the CAR IS and a rapid detach of the CAR T‐cell from the target cell. Overall, the mechanosignaling in the CAR IS initiates faster with higher intensity, while the signaling duration is shorter compared to the TCR IS.^[^ ^38^ ^]^ Such a rapid CAR signaling transduction induces fast recruitment and release of cytotoxic granules in the CAR IS,^[^ ^38^ ^]^ which enables a rapid serial cytolysis in cancer cell. Nevertheless, few studies have systematically analyzed the correlation between the rapid response of CAR T‐cell and the unique mechanotransduction in the disorganized CAR IS. Elucidation of the mechanotransduction in the nonclassical CAR IS would decipher the mechanism of the rapid responses and advantage our future CAR designs with mechanobiology‐based tools and medicines.

### 2.3 Spacing and Clustering Effect of TCR and CAR Activation

The TCR and CAR immunoreceptors require proper clustering and spacing to achieve an effective activation of the T‐cell. For instance, Manz et al. found that a TCR microcluster composed of at least four TCR–pMHC complexes would support the downstream TCR signaling and calcium influx.^[^ ^26^ ^]^ Meanwhile, an effective T‐cell activation requires an ≈200 nm cluster of TCR–pMHC complex. Such a TCR clustering was described in the kinetic‐segregation (KS) model.^[^ ^53^, ^54^ ^]^ While in the CAR T‐cell, the density of cancerous antigens has to be adequate to support the clustering and activation of CAR. Watanabe et al. prepared and tested a serial of cancer cells with the antigen density from 200 to 250 000 antigens per cell to activate CAR T‐cell (**Figure** **6A**).^[^ ^22^ ^]^ They found that an antigen density threshold around 200 molecules per cell is adequate to support the cytolysis of cancer cells, whereas initiating cytokine release from the CAR T‐cell requires around 2000 antigen molecules per cell. Therefore, the antigen density potentially dominates the CAR T‐cell cytotoxic function.

To further explore the exact nanospacing threshold of TCR ligands for effective activation, a few attempts have been made to control the geometric arrangement by immobilizing TCR binding ligands on nanoparticle arrays.^[^ ^12^ ^]^ Initially, Pageon et al. discovered that the naturally triggered CD3–TCR–pMHC nanoclusters had 55 molecules and a diameter of ≈185 nm, corresponding to an equivalent average spacing of 10 nm (Figure 6B).^[^ ^55^ ^]^ However, such a 10 nm TCR nanospacing may not represent the threshold for the effective TCR signaling activation. In a recent study by Cai et al., the nanospacing threshold of TCR has just been revealed using a gold nanoparticle array substrate (Figure 6C).^[^ ^27^ ^]^ By utilizing a sophisticated nanopatterning of anti‐CD3 antibodies, Cai et al. found that the TCR activation by anti‐CD3 is most effective on 240 nm diameter clusters with 40 nm lateral interligand spacing, and the downstream phosphorylation signaling reduces with the lateral spacing increases from 40 to 100 nm.^[^ ^27^ ^]^ Furthermore, they showed that if the axial spacing of the single activating ligand is decreased by 10 nm on the nanopatterned substrate, the inhibitory molecule CD45 can only be partially excluded, which limits the effective TCR signaling to happen only below 50 nm nanospacing.^[^ ^27^ ^]^ The precision of the nanopatterned substrate elegantly revealed the 40 nm threshold of the TCR nanospacing and the essential axial spacing to exclude the CD45 around the activating ligand clusters for effective activation.

As for the nanospacing threshold of CAR for effective activation, it is still debatable whether a threshold of CAR nanospacing is required for the CAR T‐cell activation. By quantitating the accumulation of Lck clusters and actin rings in CAR T‐cell, Davenport et al. concluded that the CAR ligands require a proper clustering and nanospacing for activation.^[^ ^38^, ^56^ ^]^ On the contrary, Sommermeyer et al. observed the CAR‐fluorescent protein fusion constructs during CAR activation and found that the CARs uniformly distribute on the activated CAR T‐cell.^[^ ^57^ ^]^ Moreover, the antigen‐independent CAR clustering may even cause the early exhaustion of CAR T‐cell with inferior antitumor efficacy in vivo.^[^ ^57^ ^]^ Nevertheless, the exact thresholds of CAR clustering and nanospacing in CAR activation or whether it is needed still remain understudied and require a further investigation. Up to date, most of these research works quantified the degree of T‐cell activation mainly by the immunofluorescent staining against the phosphotyrosine around the IS. Further research should figure out the exact molecules in the TCR and CAR signaling that are separately influenced by the lateral and axial spacings of antigen, respectively. The explorations on the antigen spacing thresholds would provide valuable information for the design of new generations of CAR T‐cells with improved antigen recognition and cytotoxic efficiency.

### 2.4 Mechanical Forces in TCR and CAR Activation

Other than the clustering and spacing of the immunoreceptors, the magnitude of the tension force transmitted through the CAR/TCR may largely determine the downstream cascades, enabling the antigen recognition and discrimination (Figure 2). To precisely detect the piconewton (pN) force in the immunoreceptor complex, Liu et al. have utilized different combinations of DNA‐based nanoprobes to demonstrate that an individual TCR–pMHC binding complex would require a force greater than 12 pN but less than 56 pN upon activation (Figure 6D).^[^ ^58^ ^]^ The magnitude of the transmitted force further increases with the sensitivity of TCR to the MHC complex. An accumulating force above threshold would boost the TCR clustering and immune responses as revealed by the enhanced ZAP70 activities around the TCR.^[^ ^59^ ^]^ While the magnitude of force influences the activation efficiency, the accumulation of tension force above the threshold requires that the generated force will not diminish as the applied force increases above the threshold in the TCR.^[^ ^60^ ^]^ Collectively, the force in a single TCR needs to be accumulated above the 12 pN threshold to trigger the biomechanical feedbacks that enhance the mechanosensing of the TCR to pMHCs, so as to achieve the successful antigen discrimination and effective immune responses.

CAR normally requires a different level of binding force to form a CAR–antigen complex. However, Chang et al. have recently modified CAR T‐cell to recognize the soluble antigen through the CAR mechanical dimerization, indicating the essential role of mechanical force in the CAR activation.^[^ ^61^ ^]^ Considering that CAR is a receptor complex includes a fragment derived from an antibody, the magnitude of the binding force required in activating CAR might be different from that of in TCR. According to that the average magnitude of binding force between a recombinant antibody and the antigen is in the range of 60–100 pN,^[^ ^62^ ^]^ we speculated that the binding force for CAR would be significantly higher than the TCR binding force range of 12–56 pN. Nevertheless, the specific binding forces and the mechanosensing thresholds of different CARs remain elusive and challenging for future studies. An accurate characterization of the binding forces in different types of CARs is an urgent need for a better design of new and efficient CARs.

### 2.5 Catch–Slip Bond Mediated Force Transmission in TCR and CAR

**Figure 4:**
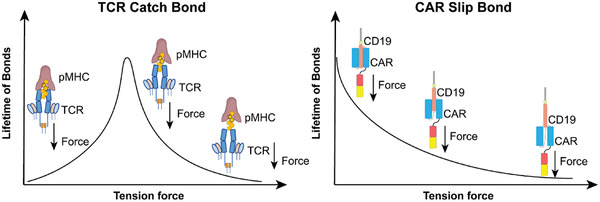
The catch and slip bonds mediated force transmission in TCR and CAR immunoreceptor–antigen complex. The different magnitudes and kinetic profiles of the force in the TCR IS and CAR IS attribute to the different nature of the bonds generated in the TCR–pMHC complex and the CAR–CD19 complex.

It is inspiring to analyze the force generation mechanisms respectively in a single TCR–pMHC and a CAR–antigen complex. Recently, researchers have identified that TCR utilizes the catch–slip bonds to recognize the target antigen with the highest binding affinity (**Figure** **4**).^[^ ^31^ ^]^ The major difference between a catch bond and a slip bond is the lifetime under an applied force. The lifetime of a catch bond would increase within an extent under the applied force, while the lifetime of slip bond would decrease. Recently, the catch–slip bond activities in the TCR–pMHC complex were systematically described by Hong, Liu, and Zhu et al. (Figure 6E).^[^ ^16^, ^31^, ^60^, ^63^ ^]^ They monitored the binding kinetics of a single TCR and a pMHC complex presenting different antigens and found that the antigen peptide regulates the bonding lifetime of TCR.^[^ ^60^ ^]^ Initially, the slip bonds are formed for all peptides right after ligation. But when the mechanical force is applied on an agonist peptide, the slip bond would transform into a catch bond, generating increasing force as the applied force rises, therefore passing on the mechanosignal to the TCR (Figure 4). However, when a force is applied to a nonagonist peptide, the previously formed slip bond remains unchanged with minimum force generation and leads to the loss of further mechanosignals in TCR. Importantly, CARs depend on the antibody‐derived fragments to bind antigen on the target cell, where a slip bond is normally exhibited (Figure 4).^[^ ^64^ ^]^ Therefore, a unique mechanotransduction mechanism, other than the catch bond mechanism in the conventional TCR–pMHC complex, may be functioning.

The conformational changes of the immunoreceptors have recently been systematically analyzed to elucidate the force generation during the antigen recognition process. A recent research has connected the catch slip bonds with the biomolecular geometrical changes in the TCR–pMHC complex. Sasmal et al. observed the conformations of individual TCR‐CD3*ζ* receptors in primary T‐cells and studied the bond distance between the ligated TCR and pMHC in situ.^[^ ^65^ ^]^ They found that the most probable intermolecular TCR–pMHC distance is around 44 ± 9 Å for the super agonist pMHC, 54 ± 11 Å for normal agonist pMHC, and 66 ± 18 Å for a weak agonist pMHC, which demonstrates that the shorter distance between TCR and pMHC favors the formation of the stronger catch bond in the TCR–pMHC complex. Moreover, previous studies have found that the dissociation rate (off‐rate) of TCR–pMHC complex decreases while the bond lifetime increases under a 10–15 pN force, indicating the formation of catch bonds.^[^ ^60^, ^66^ ^]^ To decouple the TCR binding with cellular activities during the generation of force, Limozin and Robert et al. measured the dissociation kinetics of TCR–pMHC in a cell free platform and acquired the dissociation rate of the TCR–pMHC complex under the 6–15 pN force,^[^ ^4^ ^]^ which is in consistent with the results measured from the cell system.^[^ ^60^ ^]^ Therefore, it suggests that a force on the order of 10–15 pN is required for the TCR–pMHC binding process. The agonist peptide in the pMHC would further increase the lifetime of the generated catch bonds to activate TCR.^[^ ^54^ ^]^ Interestingly, researchers did not observe the formation of any catch bond in the cell‐free platform.^[^ ^4^ ^]^ Instead, the catch bonds are only formed in the TCR connected with the cytoskeleton of live T‐cell.^[^ ^60^ ^]^ It indicates that the cytoskeleton activities are also involved in the immunoreceptor–antigen binding process and collectively induce the formation of the catch bonds during the antigen recognition.^[^ ^4^ ^]^

Collectively, the formation of catch bond in TCR is determined by the antigen type, TCR–pMHC bond distance, and cytoskeleton activities. Similarly, the formation of the force in a CAR–antigen complex can attribute to the surface antigen type, bond distance, and the cytoskeleton activities. These research works on TCR provide valuable insights for the future research on force generation in CARs. The binding in a CAR–antigen complex is less stable and more rapid than the TCR–pMHC complex.^[^ ^38^ ^]^ Therefore, novel mechanomarkers describing the rapid changing rates of CAR–antigen binding kinetics may be required to associate the mechanosensitivities of CAR T‐cell to the immune effectiveness. Besides, the force spectrum of CAR T‐cell is optimized to be monitored with real‐time microscopy with ultrahigh speed, resolution, and throughput (Figure 6F).

### 2.6 Peripheral and Distal Signaling Events in Antigen Recognition

Besides the central area of IS where CAR/TCR locates, the peripheral and distal molecules have important regulatory functions on the cytotoxic killing (Figure 3).^[^ ^68^, ^69^ ^]^ For instance, the exclusion of CD45 molecules during the CAR/TCR IS formation facilitates the downstream Lck and ZAP70 mediated phosphorylations in the KS model,^[^ ^53^, ^70^ ^]^ and ensures the close contact of immunoreceptor–antigen complex.^[^ ^71^ ^]^ Karlsson et al. showed that the CD45 exclusion and ZAP70 recruitment are required in the activation of CD19‐specific CAR T‐cell, which is similar to those of the classical TCR IS.^[^ ^70^ ^]^ Moreover, in the peripheral IS, the major signaling molecules are adhesion‐related, including LFA‐1 and talin. The engagements of these adhesion molecules play important roles in antigen recognition. First, the adhesion molecules alleviate the requirement of TCR ligand density^[^ ^72^ ^]^ and augment the tension force over the threshold by chemo‐mechanical crosstalk.^[^ ^58^ ^]^ Second, the immunoreceptor–antigen engagement would in turn increase the affinity of LFA‐1 to the intercellular adhesion molecule 1 (ICAM‐1).^[^ ^73^ ^]^ The LFA‐1 ligation would create an inside‐out mechanosignal to enhance actomyosin forces, which further increases the affinity of other integrins on T‐cell.^[^ ^74^ ^]^ The overall increased T‐cell adhesion onto the target cell stabilizes the CAR/TCR IS, providing the prolonged mechanosignals required for the effective activation. Due to the abrupt cytoskeleton structures of CAR IS, the adhesion signaling events of the CAR IS are distinct from the TCR IS in an LFA‐1‐independent manner.^[^ ^38^ ^]^ Researchers speculated that the CAR T‐cell may skip the formation of an LFA‐1‐dependent stable IS structure to induce the cytotoxic function,^[^ ^38^ ^]^ which justifies the rapid lytic granule release and cytoskeleton activities in CAR T‐cell.

### 2.7 Cytoskeletal Dynamics during the IS Formation

Despite numerous explorations on the immune receptor mechanobiology, how mechanosignals are translated into the cytoskeleton and how the cytoskeleton force affects the mechanotransduction remain largely unexplored in T‐cell. After TCR/CAR ligation with antigen, actomyosin activities synergistically induce a retrograde flow of actin in the IS and T‐cell, which generates a pulling force along the cytoskeleton (Figure 1).^[^ ^75^ ^]^ Such a retrograde actin flow‐mediated mechanical signal is essential for the maintenance of tension force in TCR–pMHC and CAR–antigen complex. During this process, T‐cell forms F‐actin‐rich protrusions, exerts synaptic forces at the IS, and coordinates the mechanical output with perforin secretion.^[^ ^17^ ^]^ These synaptic protrusions, which depended on the cytoskeletal regulator Wiskott–Aldrich syndrome protein (WASP) and the actin related protein 2/3 (Arp 2/3) to nucleate branched actin, were required for synaptic force exertion and efficient killing. Similarly, Murugesan et al. showed that activated T‐cell form numerous Arp 2/3‐mediated circular structures and formin‐mediated linear bundles in the lamellipodium at the distal IS.^[^ ^24^ ^]^ Such actomyosin polymerization motivates the spreading of T‐cell across the activating surface for maximum antigen–immunoreceptor contacts. Moreover, Fritzsche et al. observed the dense cortical network of ring‐shaped actin in the basal lamellipodium.^[^ ^64^ ^]^ Such a dense network of cytoskeleton ensures the mechanosensitivity and timely transduction of mechanosignals from the immunoreceptor. Above the dense network, there is a second layer of highly ramified actin to further transduce the mechanosignals into the nucleus. Both the ring‐shaped actin network and the ramified network contracted to reorient the cytoskeleton in the late stage of T‐cell activation. These two layers of actin serve as the structural basis of the mechanotransduction in T‐cell. Recently, a quantitative strategy based on the actomyosin activities in the IS was developed to evaluate the CAR T‐cell immune effectiveness. For example, Xiong et al. quantified the actin polymerization and the expressions of major signaling molecules in the IS of CAR T‐cell.^[^ ^76^ ^]^ They found that the actin polymerization was robust in the IS of fully activated CAR T‐cell, together with high expression of a critical CAR signaling molecule pZeta. Besides, the CD19 clustering and lytic granule activation were consequently improved as the results of enhanced actomyosin activities in the CAR IS.^[^ ^76^ ^]^

Apart from the actomyosin, microtubules are also important components of the CAR/TCR IS.^[^ ^10^, ^77^ ^]^ The microtubules serve as the scaffold to guide the reorientation of centrosome toward the immunoreceptor–antigen complex and the formation of the CAR/TCR signaling microclusters (Figure 1).^[^ ^78^ ^]^ Such a microtubule‐mediated remodeling process is essential for the release of lytic granules in central IS. During the microtubule remodeling, the movements of the centrosome and CAR/TCR microclusters generate tension force and influence the cytoskeletal dynamics. The microtubules as accompanied by the extension of actin filaments facilitate the T‐cell mechanosensing to the local rigidity of its environment and modulates the force generation during T‐cell activation and IS organization.^[^ ^10^ ^]^ Commonly, the microtubules in the IS decrease the tension force in the cytoskeleton through the Rho/Rho‐associated coiled‐coil containing protein kinase (Rho/ROCK) signaling and GTP hydrolase (GTPase) activities.^[^ ^77^ ^]^ Remarkably, the growth speed of the microtubules almost remains constant throughout the activation process, 80 ± 53 nm s^−1^ on average.^[^ ^77^ ^]^ Thus, it is the presence rather than the growth speed of the microtubules that affects the T‐cell cytoskeleton force dynamics. In fact, the microtubule growth is negatively correlated with the retrograde flow of the actin, offering an important regulation mechanism of the cytoskeleton dynamics during T‐cell mechanotransduction.^[^ ^66^ ^]^ All these results indicate the essential role of the interactions between the actomyosin and microtubule networks in T‐cell mechanotransduction.

In addition to the influence on the rate of retrograde flow, the microtubules also largely determine the killing efficiency of CAR T‐cell. The microtubules anchor the cytotoxic granules in the network and transport the granules toward the center of the IS, enabling the rapid release of lytic granules and the upcoming cytolysis of cancer cells.^[^ ^79^ ^]^ Since the release of lytic granule is faster in CAR T‐cell compared to that of the canonical T‐cell, we speculated that the microtubules in the CAR IS more dynamic than that in the TCR IS. Particular molecular mechanism that regulates the microtubule kinetics is expected to explain the potentially robust dynamics of the microtubules in the activated CAR T‐cell. Recent researches have shown that the Rab GTPases including Rab11 and Rab35 interact with the tubulin cytoskeleton to generate the force for the transportation of granules, providing an ideal target protein for the modulation of microtubule dynamics in CAR T‐cell.^[^ ^80^ ^]^ Analyzing the functions of microtubules in the mechanotransduction of CAR IS may help directly modulate the immune effectiveness of new generation CAR T‐cells.

### 2.8 Mechanotransduction at the IS Interface

**Figure 5:**
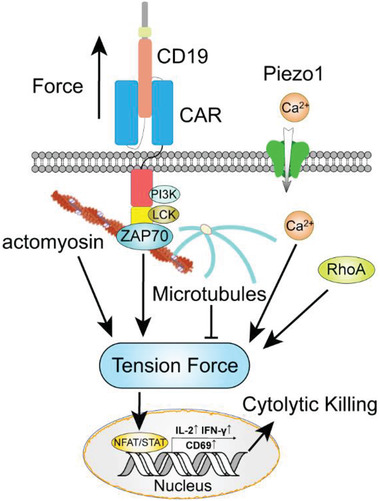
The mechanotransductive signaling pathways in CAR T‐cell.

The dynamics of the CAR‐connected actomyosins are determined by the mechanosignals from the CAR–antigen complex, which are translated through the intracellular CD28 costimulatory domains in CAR (Figures 2 and **5**).^[^ ^81^ ^]^ To analyze how the mechanosignals from immunoreceptor influence the mechanotransduction in T‐cell cytoskeleton, Colin‐York et al. photobleached a small area on the leading edge of T‐cell and monitored the actin dynamics, myosin contraction, and actin flow velocity under different activation conditions.^[^ ^82^ ^]^ Interestingly, they discovered that the rate of actin retrograde flow largely depends on the antigen unbinding kinetics. The pMHC binding with the strongest agonist ligand induced a low actin flow rate of 77.12 ± 17.9 nm s^−1^, which is significantly slower than the actin flow rate of 95.5 ± 23.3 nm s^−1^ in the nonagonist antigen. However, the peak traction force (160 pN) generated on the basal surface of contact is independent of various antigens with different affinities. Such a mismatch between the peak force and antigen affinity results from the active adjustment of traction force by the cytoskeleton. This recent work is the first research to prove that the dynamics of the actomyosin cytoskeleton actively normalize the tension force experienced by the TCR in an antigen‐dependent manner. Moreover, when they selectively hindered the myosin II activities, the retrograde flow was maintained. However, such a myosin II activity that is generally essential for the traction force generation^[^ ^83^ ^]^ is controversial in the cytoskeleton dynamics of T‐cell, which requires further investigations.

The cytoskeleton remodeling process in activated T‐cell also in turn influences the mechanotransduction at the IS interface.^[^ ^84^ ^]^ During the actin retrograde flow, the cytoskeleton applies inward forces on the immunoreceptor–antigen complex and transports them toward the central IS.^[^ ^68^ ^]^ Such a force from the cytoskeleton further amplifies the original tension and mechanosignals due to the catch‐bond nature in the TCR–pMHC complex, and potentially leads to the dissociation of intracellular domains of CAR from the cytoplasmic membrane to expose the reactive sites for the CAR signaling molecules.^[^ ^85^ ^]^ Also, the cytoskeleton force leads to the partial dissociation and translocation of CAR–antigen and TCR–pMHC complexes.^[^ ^86^ ^]^ Such a force‐induced translocation enables the single CAR/TCR to interact with multiple neighboring antigens, thereby enhancing the overall TCR signaling strength. Collectively, the CAR/TCR activation influences the dynamics of the T‐cell cytoskeleton remodeling, creating a positive feedback loop that tunes up the traction force and mechanotransduction at the CAR/TCR IS interface.

**Figure 6:**
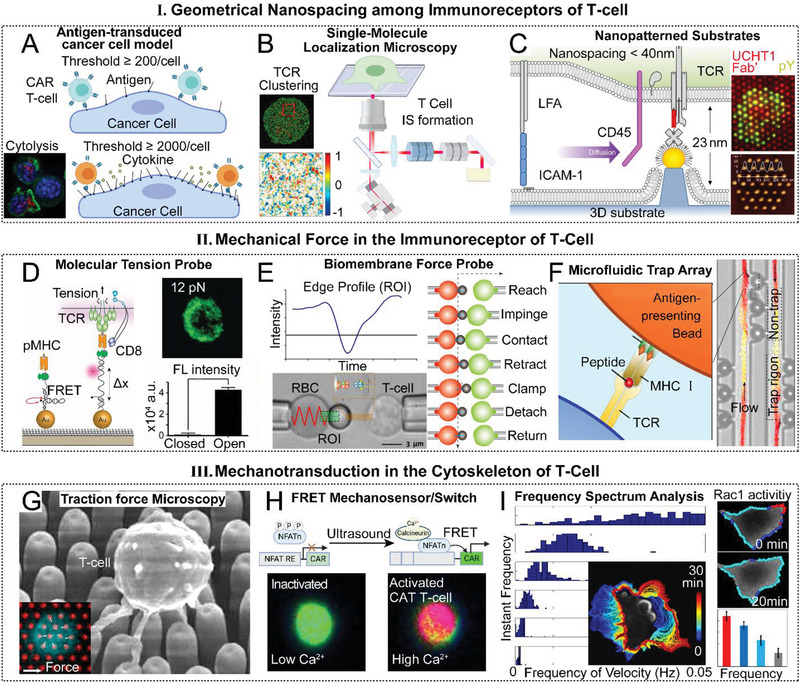
The current bioengineering strategies for deciphering the T‐cell mechanoimmunology. A) Antigen‐transduced cancer cell models with the antigen density from 200 to 250 000 antigens per cell revealed the density thresholds for cytolysis and cytokine secretion in CAR T‐cells.^[^ ^22^ ^]^ B) The single‐molecule localization microscopy has demonstrated the natural clustering and spacing of TCRs during the IS formation. Adapted with permission.^[^ ^55^ ^]^ Copyright 2016, the National Academy of Science. C) Nanopatterned gold nanoparticle array substrates revealed the lateral spacing and vertical height thresholds of the TCR ligands complex for an effective activation of TCR. Adapted with permission.^[^ ^55^ ^]^ Copyright 2018, Springer Nature. D) The DNA‐based molecular tension probe (MTP) detected the threshold of tension force generated in a single TCR–pMHC complex. Adapted with permission.^[^ ^27^ ^]^ Copyright 2016, the National Academy of Science. E) The biomembrane force probe (BFP) discovered the dynamics and catch–slip bond nature of the force in the TCR–pMHC ligation. Adapted with permission.^[^ ^63^ ^]^ Copyright 2015, the MYJoVE Corporation. F) The microfluidic trap array enabled the high‐throughput measurement of the TCR–pMHC interaction affinities. Adapted with permission.^[^ ^32^ ^]^ Copyright 2017, the American Institute of Physics. G) The PDMS micropillar array traction force microscopy (TFM) analyzed the spatiotemporal spectrum of the tension force in the cytoskeleton of activated T‐cell. Adapted with permission.^[^ ^19^ ^]^ Copyright 2016, Elsevier Inc. Adapted with permission.^[^ ^89^ ^]^ Copyright 2017, Springer Nature, respectively. H) A FRET mechanosensor and switch remotely triggers the CAR and simultaneously visualizes the calcium dynamics during the CAR T‐cell activation. Adapted with permission.^[^ ^5^ ^]^ Copyright 2018, the National Academy of Science. I) The frequency spectrum analysis showed consistent instantaneous frequency distribution rules of edge velocity and Rac1 activities in migrating cells. Adapted with permission.^[^ ^37^ ^]^ Copyright 2018, Public Library of Science.

Recently, novel force measuring platforms and algorithms have been developed to quantitatively reveal the traction force dynamics during T‐cell activation. Different from a simple pushing or pulling force, the traction force in T‐cell activated by antibodies against CD3 and CD28 would first generate an outward pushing force then followed by an inward contracted pulling force.^[^ ^37^, ^87^ ^]^ Importantly, the traction force was generated between anti‐CD3 antibody and CD3–TCR complex, while the costimulatory CD28 enhanced the force strength by activating PI3K signaling (Figure 6G).^[^ ^88^, ^89^ ^]^ In another study, Husson et al. activated T‐cell with antibodies against CD3 and CD18 to measure the cytoskeleton dynamics of the activated T‐cell.^[^ ^78^ ^]^ They found that T‐cell would first exert a 24 pN pushing force against the activating bead within the 140 s after anti‐CD3–TCR ligation, followed by a gradual 16 pN inward pulling force from 180 s. The actin polymerization‐induced force loading rate of pulling force linearly increases with the stiffness of the antigen‐bearing microbead, indicating the mechanosensing nature of the IS.^[^ ^10^, ^88^ ^]^ Collectively, these researches potentially explain the general contribution and pattern of mechanical force transduction during T‐cell activation. The outward traction forces enable the spreading of lamellipodium and simultaneously increase the antigen‐receptor contacts in the IS, while the inward pulling forces transfer the extracellular mechanosignals through the CAR/TCR immunoreceptors. To date, little research has been conducted on CAR T‐cell mechanotransduction, leaving the force transmission pattern in CAR T‐cell undefined. Since CAR shares the same CD3 and CD28 stimulatory domains with TCR, the findings from conventional T‐cell may also be applicable to CAR T‐cell. However, given the distinct structure of CAR from TCR, CAR T‐cell mechanotransduction might follow different dynamic patterns, even among different types of CAR T‐cells.

### 2.9 Mechanotransduction in the Nucleus of T‐Cell

Accumulating evidences have shown that the mechanotransduction from the cytoskeleton into the nucleus would regulate the CAR/TCR activation and IS formation. Researchers have found that the transduction of tension force into the nucleus relies on the “linker of nucleoskeleton and cytoskeleton (LINC)” complex.^[^ ^91^ ^]^ The nucleus directly responds to the mechanical signals with geometrical deformations and alters gene expression through Nuclear factor of activated T‐cells (NFAT) and Janus kinase/signal transducers and activators of transcription (JAK/STAT) signaling cascades (**Figure** 5).^[^ ^92^ ^]^ To analyze the endogenous nuclear actin network of T‐cell, Tsopoulidis et al. combined the super‐resolution microscopy and computational segmentation to demonstrate that the mechanotransduction causes a remodeling in nuclear actin of T‐cell.^[^ ^93^ ^]^ Just like in the cytoskeleton, there is a sharp increase of Ca^2+^ influx into the nucleus, and Arp2/3 complex mediates the rapid formation of a dynamic and mechanosensitive nuclear actin network. An increase in nuclear stiffness would decrease the nuclear mechanosensitivity and hinder the effector functions of CD8+ T‐cell.^[^ ^81^ ^]^ The mechanosensing of the nuclear actin network partially explains the effects of environment stiffness on the immune functions. In a recent study, Saitakis et al. cultured T‐cells on polyacrylamide (PA) hydrogels of varying stiffness and discovered that the expression of immune‐reactive genes is potentiated by the increased stiffness. They speculated that lamins would transduce the mechanosignals into the nucleus and thus alter the gene expressions.^[^ ^82^ ^]^ As a support of this speculation, the expression of nuclear lamin‐A has been previously reported to increase the downstream target gene expressions, and accelerate the formation of mature IS.^[^ ^95^ ^]^ All these findings demonstrate the potential role of nucleus to regulate the T‐cell cytotoxic functions. Considering the high similarity of nucleus in normal T‐cell and CAR T‐cell, the mechanotransduction in the nucleus would provide an effective approach to modulate the gene expression in CAR T‐cell. An accurate mechanobiological modulation of nucleus might serve as a sensitive brake to precisely control the cytotoxic effects of CAR T‐cell in the clinical trials.

### 2.10 Mechanosensitive Ion Channel Piezo1 in T‐cell Activation

There has been an increasing number of ion channels found to react to the mechanocues, which play important regulatory roles in T‐cell activation.^[^ ^96^ ^]^ Among them, the mechanosensor Piezo1 is becoming increasingly important in the mechanobiology research of CAR T‐cell (Figure 5). Piezo1 is a mechanically activated ion channel that is highly conserved across cells.^[^ ^97^ ^]^ Recent research has revealed that Piezo1 contributes to T‐cell activation by inducing calcium influx.^[^ ^98^ ^]^ During the process of antigen recognition, Piezo1 has been involved in amplifying the TCR/CAR signaling.^[^ ^99^ ^]^ Furthermore, Pan et al. have developed a mechanogenetics system to use ultrasound tweezers to remotely yet precisely manipulate CAR T‐cell activation by motivating Piezo1 mechanosensor (**Figure** **6**H),^[^ ^5^ ^]^ demonstrating the utilization of mechanotransduction in controlling CAR T‐cell cytotoxic activities. Furthermore, Nonomura et al. have found that the mechanical and chemical stimulation of Piezo1 induced a calcium‐dependent activation of the nuclear factor of activated T‐cell.^[^ ^100^ ^]^ Similarly, Liu et al. showed that the Yoda1‐mediated overactivation of Piezo1 leads to a 250% elevation of CD69 gene expression in T‐cell upon activation, and the siRNA‐mediated Piezo1 deficiency leads to the impaired T‐cell activation, accompanied by the abrogation of calcium influx.^[^ ^98^ ^]^ As a second messenger, the accumulating calcium ions will trigger the activities of numerous calcium‐depending enzymes including the calpain, a universal cystine protease interacting with many actin‐binding proteins.^[^ ^101^ ^]^ The calpain‐mediated disassembly of talin regulates the dynamics of the actomyosin cytoskeleton and microtubules in the T‐cell.^[^ ^102^ ^]^ Therefore, Piezo1 extensively influences the actomyosin dynamics in a calcium‐dependent manner. The unrevealed regulatory roles of Piezo1 and other mechanosensors would greatly inspire the development of new generation CAR T‐cells.

### 2.11 Mechanoregulation of Metabolism and Genetics in T‐Cell Activation

The CAR/TCR activation triggers extensive cytoskeleton remodeling and impacts on various organelles and chromatins, which are reflected in the alteration of metabolic status of the T‐cell. The metabolism of T‐cell determines the progression of the immune responses. The naïve T‐cell normally possesses fatty acid metabolism with low rates, which need to be remarkably upregulated upon activation.^[^ ^103^ ^]^ Activation of naïve CD8+ T‐cells triggers a rapid metabolic switch and phenotypic transformation. Essentially, both glycolysis and glutaminolysis are enhanced to meet the enormous energetic demands for the rapid proliferation, force generation, signaling transduction, and production of cytolytic granules in the activated T‐cell.^[^ ^103^ ^]^ Emerging studies have revealed the potential mechanoregulation mechanisms of the metabolism in T‐cell activation. For instance, CD28 costimulation has been reported to augment the mechanosignals in TCR/CAR through the PI3K signaling.^[^ ^24^ ^]^ Recently, Beckermann et al. have further proved that CD28 costimulation effectively elevates the glycolysis and mitochondrial oxidative metabolism of the activated CD8+ T‐cell through the upregulated expression of glucose transporter 3 (GLUT3).^[^ ^104^ ^]^ Meanwhile, the GLUT expression can be induced through the PI3K/Akt signaling.^[^ ^105^ ^]^ Therefore, the mechanosignals from TCR/CAR may induce high metabolism rates and mitochondrial activities to fulfill the effector functions.

Meanwhile, the force generation and metabolism upregulation may also induce profound genetic changes in the activated T‐cell. The rapid metabolic reprogramming and phenotypic transformation in T‐cell activation are associated with markedly increased expressions of numerous genes, including the *IL2RA*, *IL2RB, HIF1A*, *IRF1*, and *MYC*, which play key roles in activation‐induced metabolic reprogramming across multiple pathways to augment the T‐cell activation.^[^ ^103^ ^]^ The mechanoinduced oxidative metabolism produced various metabolic enzymes like pyruvate kinase and metabolites like oxidized nicotinamide adenine dinucleotide (NAD^+^),^[^ ^94^ ^]^ all of which participate the histone acetylation and chromatin remodeling and regulate the consequent cell behaviors and T‐cell cytokine secretion. The latest genetic studies have discovered close interactions among mechanoimmunology, metabolism, and genetic alterations, creating an understudied field to analyze the potential mechanoregulation of the genetics throughout the T‐cell activation. Elaborating the short‐term and long‐term influences of mechanotransduction on the transcriptional programs of chromatins in different phenotypes of T‐cells would potentially predict the therapeutic efficiency of CAR T‐cell therapy. For instance, recent research utilizing the ATAC‐seq (assay for transposase‐accessible chromatin using sequencing) technique demonstrated that effector, memory or exhausted phenotypes were associated with characteristic changes in chromatin accessibility away from the naive T‐cell state.^[^ ^95^ ^]^ Particularly, the exhausted T‐cell suffering from decreases in the mechanosensitivity and mechanotransduction upon activation, exposed additional editable chromatin sites which lead to the overexpression of PD‐1 and the inhibition of the effector functions.^[^ ^107^ ^]^ Yet, the potential interconnections between the retarded mechanotransduction of the exhausted T‐cell and the alteration in the chromatin accessibility require a further exploration. With the development of novel genetic analytic techniques including the microarray, RNA sequencing, and transcriptome network analysis, the interactions among the T‐cell mechanotransduction, metabolism, and genetic alterations will provide valuable insights for the development of new and better immunotherapies for cancer as well as the metabolic and genetic diseases.

## 3 Current Bioengineering Strategies for Deciphering T‐Cell Mechanoimmunology

### 3.1 Advanced Microscopy for T‐Cell Cytoskeleton Analysis

The development of advanced microscopy with super‐resolution has unveiled a variety of essential actomyosin structures during the IS formation (Figure 6B, Table 1). Initially, the total internal reflection fluorescence (TIRF) microscope was developed to provide high resolution and accurate tracking of the single fluorophores. TIRF technique is especially suitable for imaging the interface between the cell and substrate and therefore been utilized to visualize the dynamic assembly of the immunoreceptors at the IS interface.^[^ ^23^ ^]^ Moreover, to acquire a high intracellular resolution, Murugesan et al. utilized a structured‐illumination microscope (SIM) to show that activated T‐cell form numerous Arp2/3‐mediated circular structures and formin‐mediated linear bundles.^[^ ^24^ ^]^ Such a microscope greatly eliminates the background fluorescence noise and reveals the delicate actin structures with ultrahigh resolution. Similarly, Fritzsche et al. further utilized the super‐resolution stimulated emission depletion (STED) microscopy and lattice light‐sheet microscopy (LLSM) to completely record the dense cortical network of ring‐shaped actin in the basal lamellipodium.^[^ ^25^ ^]^ 3D reconstructions of the STED microscopy image have unveiled a previously unreported actin structure with important mechanotransductive functions. The advanced microscopy techniques with super resolution will disclose the structural basis of the CAR IS formation and CAR T‐cell activation in future research.

**Table 1:** A brief summary of the novel bioengineered strategies exploring T‐cell mechanoimmunological mechanisms

Research objectives		Bioengineering strategies	Applications in mechanoimmunology research
I. Imaging/probing IS structure	Antigen density threshold	Antigen‐transduced cancer cell model	Reveal the antigen threshold of cytolysis and cytokine secretion in CAR T‐cell^[^ ^22^ ^]^
	Cytoskeleton structures	Total internal reflection fluorescence (TIRF)	Visualize the dynamics of immunoreceptors at the IS interface^[^ ^23^ ^]^
	Cytoskeleton structures	Structured‐illumination microscope (SIM)	Reveal the circular structures and linear bundles of actin in IS^[^ ^24^ ^]^
	Cytoskeleton structures	Super‐resolution stimulated emission depletion (STED) microscopy	Visualize the cortical actin networks of the activated T‐cell^[^ ^25^ ^]^
	Cytoskeleton dynamics	Lattice light‐sheet microscopy (LLSM)	Record the cytoskeleton dynamics with ultrahigh spatiotemporal resolution^[^ ^25^ ^]^
	TCR/CAR nanospacing	Nanopatterned substrates	Analyze the ligand geometrical effects on TCR/CAR activation^[^ ^26^, ^27^ ^]^
	3D mechanotransduction mechanism	3D scaffold metrices	Analyze the biophysics of the IS mechanotransduction in the 3D context^[^ ^28^ ^]^
II. Measuring force in the immunoreceptor–antigen complex	Force in TCR/CAR	DNA‐based molecular tension probe (MTP)	Measure the piconewton force thresholds in activated TCR/CAR^[^ ^29^ ^]^
	Force in TCR/CAR	Single molecule atomic force microscopy (AFM)	Capture the static topography and dynamic force spectrum of the single molecules^[^ ^30^ ^]^
	Force dynamics in TCR/CAR	Biomembrane force probe (BFP)	Detect the catch–slip bonds in the mechanotransduction of TCR/CAR^[^ ^31^ ^]^
	Binding affinity of TCR–pMHC	Microfluidic trap array	High‐throughput measurement of the CAR/TCR‐antigen affinity^[^ ^32^ ^]^
	High‐throughput screening	Microfluidic immune organs‐on‐chip	Analyze the strength and persistence of the T‐cell mechanosensing in the immune organs^[^ ^33^ ^]^
III. Measuring force in the cytoskeleton	Force in the cytoskeleton	Micropillar traction force microscopy (TFM)	Measure the force dynamics of cytoskeleton^[^ ^34^ ^]^
	Force in the cytoskeleton	Hydrogel traction force microscopy	Analyze the effect of stiffness on the force dynamics of cytoskeleton^[^ ^35^ ^]^
	Force and signaling molecules in cytoskeleton	FRET mechanosensor/switch	Visualize the mechanosignals in T‐cell,^[^ ^36^ ^]^ switch on cytotoxic function of CAR T‐cell^[^ ^5^ ^]^
	Kinetics of force, motility, and molecules	Frequency spectrum analysis	Analyze the morphogenesis and mechanosensitivity of cells^[^ ^37^ ^]^

### 3.2 Nanopatterned Substrates for Probing the TCR/CAR Nanospacing and Clustering Effects

To quantitatively analyze the influences of ligand nanospacing on the TCR/CAR immunoreceptor activation, novel precisely nanopatterned substrates have been fabricated to probe TCR/CAR nanospacing and clustering (Figure 6C). To measure the quantity threshold of TCR–pMHC in a single microcluster, Manz et al. performed TCR clustering titration experiments based on a supported lipid bilayer system portioned with grids of chromium nanobarriers, which effectively limits the number of pMHC ligands available to a single TCR cluster.^[^ ^37^ ^]^ With the same approach, the antigens available to the single CAR can be carefully controlled to measure the quantity threshold of CAR in the IS. Besides the 2D nanopatterned substrates, 2.5D substrates with well‐controlled both lateral and vertical nanoscale features can enable the fine tuning of the surface topography that better mimics the complexity of IS interface and microenvironments encountered by CAR T‐cell.^[^ ^108^ ^]^ To reveal the nanospacing threshold of immunoreceptor ligands, Cai et al. successfully controlled the lateral and vertical nanospacing of the TCR activating ligands on a gold nanoparticle array substrate.^[^ ^27^ ^]^ By replacing the TCR activating ligands into the agonist antigen for the CAR, the debatable nanospacing thresholds of the CAR activation can be answered with convincing proof. Moreover, these 2D and 2.5D nanotopographic substrates can be integrated within a 96‐well plate to serve as a high‐throughput mechanobiology screening platform.^[^ ^109^ ^]^ Such a platform is compatible with high‐throughput liquid handling, high‐resolution imaging, and multiwell plate‐based instrumentation, and as well as the cell analysis techniques such as enzyme‐linked immunosorbent assay (ELISA) and flow cytometry analysis. This nanotopography and 96‐well plate‐based mechanobiology screening platform thus can potentially reveal the complex influences of substrate mechanical properties, surface topography, and biochemical factors on T‐cell activation and functional cytokine production, a result with direct clinical applicability in adoptive immunotherapy.

### 3.3 3D Scaffold and Organoid Platforms for T‐Cell Mechanoimmunology Study

Currently, our understanding of how the engineered T‐cell interact with the mechanically complex immune microenvironment is still inadequate. Considerable efforts have been made to mimic the 3D scaffold platforms to analyze the biophysics of the IS mechanotransduction. Recently, the 3D alginate scaffold matrices with constant microporosity and tunable elasticity from 4 to 40 kPa were utilized to study how the T‐cell activation is modulated by the 3D mechanical microenvironment.^[^ ^28^ ^]^ Different from the conventional 2D substrates, where a low stiffness promotes the immunoreceptor clustering and T‐cell activation, a mechanically stiffer 3D scaffolds better promote the proliferation, migration and T‐cell activation,^[^ ^28^ ^]^ indicating that distinct mechanosensing mechanisms are adopted in the context of 2D and 3D microenvironments.

The emerging 3D engineered platforms facilitate the design of synthetic lymphoid organoids by creating a favorable microenvironment resembling the natural immune tissue niche. The primary lymphoid organoids like thymus focus on the generation and differentiation of T‐cell, while the secondary lymphoid organoids like lymph nodes and spleen stress on the antigen presentation and T‐cell activation.^[^ ^110^ ^]^ A lymph node organoid scaffold made from the mesoporous silica microrods functionalized with pMHC, anti‐CD3, and anti‐CD28 antibodies effectively enhanced the activation and persistence of the embedded CD8+ T‐cells.^[^ ^111^ ^]^ By replacing the activating ligands and cytokines, such a platform can be used for the CAR T‐cell study. Based on the impacts of ECM on T‐cell, a novel lysyl oxidase (LOX) target strategy was used to modulate the mechanotransduction and clinical efficiency of T‐cell by altering the mechanical properties of the microenvironments which can be extensively adopted in various in vivo and ex vivo platforms.^[^ ^112^ ^]^ By developing these 3D scaffolds and organoid systems and protocols, we can gain valuable insights on the CAR T‐cell mechanoimmunology that are truly physiologically relevant.

### 3.4 Molecular Tension Probe for Force Detection in TCR–pMHC and CAR–Antigen Complexes

To accurately monitor the piconewton‐level forces generated in single TCR–pMHC and CAR–antigen complexes, the detection resolution of the force sensors needs to be extremely high. In the preliminary trials with atomic force microscopy (AFM) tips coated with anti‐CD3*ε* antibody or pMHC, Hu et al. detected the 500 pN pushing force and 1500 pN pulling force after T‐cell activation.^[^ ^87^ ^]^ By changing the contact time intervals of the AFM tips, they revealed the minimum contact time of 10 s for effective T‐cell activation. To achieve higher sensitivity, a single molecule AFM was used to capture the static topography of single molecules in a unique peak force tapping (PFT) scan mode.^[^ ^30^ ^]^ Besides, the dynamic conformational changes of the single molecules during the CAR–antigen ligation can be recorded by the high‐speed AFM, which potentially explains the detailed dynamics of the CAR activation. To further explore the thresholds of traction forces in TCR–pMHC ligation, Liu et al. have developed novel biomolecule‐based force measuring molecular tension probes (MTP) to greatly improve the measurement resolution to the millisecond and single biomolecule level (Figure 6D).^[^ ^29^ ^]^ By designing the base pairs and the conjugation sites of fluorophore probes on the DNA tension probes, the tension force can be precisely adjusted from 5 pN (unzipping mode) to above 50 pN (shearing mode).^[^ ^104^ ^]^ Such a DNA hairpin MTP can accurately detect the threshold of force transmitted in a TCR–pMHC complex for a successful activation. As the binding force between CAR and antigen would be the range of 60–100 pN,^[^ ^62^ ^]^ which is significantly higher than the TCR binding force, an adjusted force detection range and the stabilization of rapid generation of mechanical signals are essential in the analysis of forces in CARs with high dynamicity.

The transmission of forces involved in the CAR/TCR antigen recognition are often weak and infrequent with short duration,^[^ ^26^ ^]^ Therefore, it is challenging to analyze these rapidly changing forces due to the limited temporal resolution of current force detection probes and platforms. Recently, Ma et al. optimized a DNA hairpin MTP to detect the short‐lived force transmitted by low affinity antigens and low abundant coreceptors during the TCR activation.^[^ ^113^ ^]^ They stabilized the force activated MTP by adding a locking oligonucleotide that irreversibly binds to the activated MTP after the mechanical stimuli from the low abundance ligands. Such an irreversibly binding keeps the fluorescent signals constantly on for the detection, thereby greatly enhancing the signal intensity compared to the original real‐time MTP fluorescent signals. The intensity of the fluorescent signals was elevated by 180 times on average compared to the original strength.^[^ ^113^ ^]^ The detecting thresholds of such single molecular MTPs can be optimized for the detection of tension force in the CAR–antigen recognition.

### 3.5 Biomembrane Force Probe for Probing Force in TCR and CAR

To better understand the dynamics and nature of the force during the TCR–pMHC ligation, scientists fabricated the biomembrane force probe (BFP) with a micropipette‐held biotinylated red blood cell (RBC) conjugated with a streptavidin‐coated bead bearing the pMHC to monitor the binding kinetics of a single TCR–pMHC complex presented with different antigens (Figure 6E).^[^ ^19^, ^31^, ^60^, ^90^, ^114^ ^]^ Generally, a pulling force was applied to the TCR through the micropipette‐held RBC‐bead construct. The temporal spectrum and lifetime of the force in a single TCR will be calculated based on the displacement of the bead. By increasing the micropipette suction force on the RBC, the RBC membrane will be stretched tightly, limiting the mobility of antigen‐binding bead, therefore upregulating the mechanical signals sensed by the TCR.^[^ ^60^ ^]^ Recently, Chen and co‐workers have improved the stability of BFP system to measure the ultralong binding kinetics of PD‐1‐antibody ligation.^[^ ^115^ ^]^ By introducing a proximal reference bead on the RBC micropipette, the drifting displacement of RBC during the measurement can be monitored in real time. To eliminate the drift, a compensatory movement will be made on the micropipette clamping the T‐cell to keep accuracy in the force measurement.^[^ ^115^ ^]^ Such a real‐time feedback system effectively improves the stability of the BFP, enabling the detection of long lifetime binding kinetics between an antibody and its antigen. Similarly, researchers can present antigens with different affinity to the CAR T‐cell through such a reinforced BFP, and the spectrum of generated force in a CAR may be revealed by monitoring the lifetimes of tension forces triggered by various antigens in a single CAR–antigen complex. Furthermore, the BFP platform has been used to analyze the force dynamics in an activated T‐cell.^[^ ^90^ ^]^ By conjugating the antibodies against CD3 and CD18 on the microbead, the BFP successfully activated the TCR and LFA‐1 and triggered the mechanotransduction in the cytoskeleton. With such an approach, the magnitude and loading speed of tension force can also be revealed in CAR T‐cell.

### 3.6 Microfluidic Measurement of Immunoreceptor–Antigen Interactions

To improve the throughput of the tension force measurement, Stockslager et al. designed a microfluidic trap strategy to measure the TCR–pMHC interactions (Figure 6F).^[^ ^32^ ^]^ They fabricated a long serpentine channel in the microfluidic device and immobilized pMHC‐coated beads in hydrodynamic traps proximal to the channel. When a T‐cell was loaded in the microfluidic channel, the flowing velocity of the T‐cell would be continuously recorded as they flow by the hydrodynamic traps. Due to the interaction with the pMHC‐coated beads, the greater reduction in the velocity will reflect the higher TCR–pMHC affinity and binding force. The microfluidic device successfully enabled 24–30 cell‐bead interactions per minute on a single T‐cell, with over 76.2% of the loaded T‐cells being responsive to the pMHC ligation.^[^ ^32^ ^]^ The abnormal immunoreceptor–antigen affinity would lead to the malfunctions of these cytotoxic T‐cells.^[^ ^46^, ^116^ ^]^ Such a high throughput measurement of the TCR–pMHC and potentially the CAR–antigen binding affinities can help for the screening of most effective T‐cells.

Another microfluidic platform can be used for the mechanoimmunology research is the immune organs‐on‐chip, which can provide a more physiological relevant microenvironment ex vivo. For example, the microfluidic lymph node‐on‐a‐chip provides a biomimetic platform to study how the mechanical signals in the lymph node affect APC‐T‐cell interactions.^[^ ^33^ ^]^ Such a lymph node‐on‐a‐chip study revealed that the strength and persistence of the mechanosensing in the lymph node mostly depend on the shear stress applied to the T‐cell, and the persistence of APC‐CD8+ T‐cell interactions are much shorter than the CD4+ T‐cell.^[^ ^117^ ^]^ Other in vitro microfluidic‐based cancer immune microenvironment models, such as glioblastoma‐on‐a‐chip, have been developed to study the tumor–T‐cell interactions in immunotherapy.^[^ ^118^ ^]^ Similarly, the measurements of the affinity and mechanotransduction in CAR–antigen complexes and the real‐time monitoring of CAR T‐cell–tumor interactions in a biomimetic environment ex vivo with novel microfluidic‐based microphysiological systems would facilitate the rapid screening of the CAR T‐cell subsets with the appropriate mechanical properties and highest cytotoxicity to greatly improve the therapeutic efficiency of the CAR T‐cell‐based immunotherapy.

### 3.7 Traction Force Microscopy for T‐Cell Force Analysis

To analyze the spatiotemporal spectrum of the tension force at the CAR/TCR IS interface, researchers have developed novel traction force microscopy (TFM) platforms (Figure 6G).^[^ ^118^ ^]^ These TFM platforms share the similar principles of measurement and record the spatiotemporal traction force spectrum by utilizing time‐lapse imaging techniques. Researchers conduct biophysical analysis on the direction and magnitude of forces during T‐cell activation to acquire the force spectrum. For example, a TFM made from a densely packed array of PDMS micropillars coated with antibodies against CD3 and CD28 were used to measure the traction force dynamics during the T‐cell activation.^[^ ^10^, ^34^ ^]^ In addition to the function for force measurement, TFM platforms can also serve as substrates with tunable rigidity to study the mechanosensing of T‐cell by carefully controlling the heights and diameters of the PDMS micropillars.^[^ ^10^ ^]^ Moreover, by utilizing a TFM made from the PA hydrogel embedded with fluorescent microparticles, Hui et al. demonstrated the mechanoresponsiveness of Jurkat T‐cell toward the mechanical stiffening substrates.^[^ ^35^ ^]^ However, these 2D TFM platforms can only reveal the planar traction force parallel to the CAR/TCR IS interface on manmade substrates. Novel 3D TFM platforms are in an urgent need to in situ track subcellular forces in at a real T‐cell–tumor cell IS interface.

### 3.8 Single‐Molecule Fluorescence Resonance Energy Transfer (FRET) Mechanosensors for Visualizing Dynamic Cytoplasmic Forces

FRET‐based mechanosensors have been highlighted for their capacity of probing spontaneous dynamics of subcellular forces and mechanosensitive proteins in live cells and the compatibility of applications in 3D settings (Figure 6H). For the applications in T‐cell mechanoimmunological study, researchers have developed a genetically encoded spectrin‐repeat stress‐sensitive FRET cassette (sstFRET) mechanosensors to in situ visualize the cytoskeleton force dynamics.^[^ ^36^ ^]^ These single‐molecule sstFRET mechanosensors would provide valuable information of the subcellular force and molecular activities in mechanotransduction including actinin, RhoA, or Ca^2+^.^[^ ^119^ ^]^ To monitor the mechanotransduction events in TCR IS formation, Wan et al. have developed a broadly applicable Lck FRET biosensors containing an Lck‐sensitive tyrosine peptide to visualize the dynamic Lck kinase activities in activated T‐cell, which revealed the biophysical basis underlying TCR activation.^[^ ^120^ ^]^ In addition to serving as a mechanosensor, FRET can also serve as a mechanosensitive switch tool with pulsed ultrasound^[^ ^5^ ^]^ or light^[^ ^121^ ^]^ stimulations to noninvasively control gene activation and activate inducible CAR T‐cells for precision cancer immunotherapy. Such FRET probes showed a great promise in visualizing and modulating the activation of CAR T‐cell with a high spatiotemporal resolution, which is highly compatible with the rapid change of mechanotransduction events during the CAR T‐cell activation and tumor cytolysis.

### 3.9 Frequency Spectrum Analysis of Morphogenesis and Mechanical Signaling

Activated CAR T‐cell undergoes a rapid spatiotemporal remodeling of cytoskeleton and force during the IS formation and cytotoxic process. A real‐time monitoring and accurate characterization of the instantaneous mechanical kinetics and signal transmission in the CAR T‐cell rely on the advanced computational segmentation and analysis methods. Recently, an important mechanokinetic marker has been revealed as the instantaneous frequency spectra of the subcellular morphogenesis and mechanical signaling (Figure 6I).^[^ ^37^ ^]^ By monitoring the moving velocity of the cell edge and calculate the changing rates of the velocity, Ma et al. found that the spontaneously migrating cells show remarkably consistent instantaneous frequency distribution even the mobility is significantly heterogeneous. Furthermore, by dynamically visualize the fluorescent RhoA activities using a FRET mechanosensor, Ma et al. also monitored the spatiotemporal change of the mechanosensitive RhoA signaling in the cell during the morphogenesis process.^[^ ^37^ ^]^ Such a frequency spectrum analysis on subcellular morphogenesis and mechanosensitive signaling molecules may offer a highly sensitive and accurate classifier to monitor the rapid changes of spatiotemporal morphogenesis, force dynamics, and mechanosignaling during the CAR activation, IS formation, and cytotoxic process, so as to distinguish the subtypes of CAR T‐cells with different cytotoxic efficiency based on the cells’ intrinsic mechanosensitivity.

## 4 Conclusions

The mechanical forces exerted through the IS interface between the CAR T‐cell and the target cell is indispensable for committing cytotoxic functions. There have been increasing researches on the mechanoimmunology of the CARs, conducted from the molecular, cellular, and clinical levels. Novel technologies exploring the mechanobiological mechanisms for CAR T‐cell activation and tumor cytolysis are crucial for advancing the efficiency of immunotherapy. The rapidly evolving microscopy and force measurement technologies enable scientists to measure the piconewton force generated in the CAR IS and depict the disrupted cytoskeleton of activated CAR T‐cell. The discovery of key mechanomarkers would reveal the structural basis and molecular mechanism of the mechanotransduction in the IS of CAR T‐cell. These mechanomarkers include antigen affinity, catch slip bond, cytoskeleton retrograde flow, tension force kinetics, frequency spectrum, general mechanosensors, etc., which would better decipher the outcomes of cytotoxic CAR T‐cell in the immuno‐oncology. Bearing this in mind, new force measuring platforms need to be elevated in the resolution of force detection, sensitivity to the mechanosignals, and the throughput of detection. Similar to the biochemical markers utilized in the discovering of new drugs, the identified mechanomarkers and signals in CAR T‐cell can also be used as new targets for the development of novel mechanomedicines to improve cancer immunotherapy performance. To augment the dynamic ligation of the immunoreceptors, the feedback from cytoskeleton to the immunoreceptors can be potentially enhanced by Jasplakinolide, an commonly used actin‐stabilizing drug.^[^ ^122^ ^]^ However, the administration of Yoda‐1 would increase the sensitivity of Piezo1 with enhanced calcium influx^[^ ^97^ ^]^ and potentially further increase the traction rates and efficiency of CAR T‐cell.

Although many researches have studied the mechanoimmunology of canonical T‐cell, whether/how these findings and lessons could be adapted from classical cytotoxic T‐cell to the engineered CAR T‐cell remain challenging, since the CAR T‐cell has distinct IS structure and molecular dimensions of the CAR–antigen complex. The nanospacing thresholds, the binding kinetics, and the cytoskeleton dynamics of the activated CAR T‐cell remain largely unexplored. Furthermore, the new generations of CAR T‐cells which contain multiple stimulatory domains may further augment the mechanotransduction in the CAR through chemo‐mechanical crosstalk. Thus, it is in an urgent need to analyze how the special mechanosensing structures and mechanotransduction in CAR IS regulate the CAR T‐cell activation and the rapid serial cytotoxic killing of cancerous cells. We envision that the deepening understanding of mechanotransduction in the CAR T‐cell and the development of highly sensitive mechanoimmunological analysis platforms would altogether shed light on the design of the future generations of safe yet efficient CAR T‐cells.

## Conflict of Interest

The authors declare no conflict of interest.
